# Sensory Profile of Children and Adolescents with Autism Spectrum Disorder and Tip-Toe Behavior: Results of an Observational Pilot Study

**DOI:** 10.3390/children9091336

**Published:** 2022-09-01

**Authors:** Giulio Valagussa, Giulia Purpura, Alessandra Nale, Rita Pirovano, Miryam Mazzucchelli, Enzo Grossi, Cecilia Perin

**Affiliations:** 1 Autism Research Unit, Villa S. Maria Foundation, Tavernerio, 22100 Como, Italy; 2 School of Medicine and Surgery, University of Milano Bicocca, 20900 Monza, Italy

**Keywords:** autism spectrum disorder, toe walking, sensory impairment

## Abstract

Atypical sensory processing is frequently reported in persons with autism spectrum disorders (ASD), and it is one of the described diagnostic criteria for ASD. There is also mounting literature supporting the presence of motor impairments in individuals with ASD. Among these motor signs, tip-toe behavior (TTB) is a possible clinical finding, but its etiology is not clearly understood. It is suggested that TTB in ASD could be a sign of a sensory modulation impairment, but evidence is lacking and controversial. The main aim of this pilot study is to explore sensory features in a sample (4 females; 28 males) of children and adolescents with ASD (age range: 7–18). All participants also presented Intellectual Disability. Participants were divided in two groups, matched for age and gender, on the basis of the presence or absence of TTB (16 ASD TTB group vs. 16 ASD NO-TTB group) and then evaluated by using the Short Sensory Profile. We found that both ASD groups tend to significantly present sensory-related behavioral symptoms, but ASD TTB individuals more frequently showed the specific pattern of “under responsive/seeks sensation” than ASD NO-TTB individuals. These preliminary findings support that sensory-motor features might be taken into consideration when rehabilitation for TTB in children and adolescents with ASD is necessary.

## 1. Introduction

Autism spectrum disorder (ASD) is a complex neurodevelopmental disorder affecting 1 in 44 children [1] and resulting in an impairment of socio-communicative interaction, restricted interests, and repetitive behavior [2]. Atypical sensory processing is frequently demonstrated in individuals with ASD [3,4], and it is now listed as one possible manifestation of stereotypic and repetitive behaviors in the diagnostic criteria for ASD according to the Diagnostic and Statistical Manual of Mental Disorders 5th ed. [2]. Specifically, different profiles of sensory processing can be described in children as sensory over-responsivity (i.e., increased response reactivity to sensory input), sensory under-responsivity (i.e., decreased response to sensory input that usually stimulates a response), and sensory seeking (i.e., intensified interest for sensory stimulation) [5]. All these sensory responses often have an important influence on daily life activities (for a review, see Dunn et al. 2016) [6], and they are expressed among individuals with ASD with a large heterogeneity, suggesting a key role in the functioning of this population [7]. In 2014, Lane et al. stated that sensory subtypes in children with ASD are not well explained by other variables such as age, gender, IQ, and autism symptom severity, and appear to provide one means of classifying individuals with ASD [8]. Sensory modulation symptom severity is a reliable means of classifying variance within children with ASD [9]. For these reasons, given the importance of these aspects in the clinical setting of children with neurodevelopmental disorders, a systematic review aimed to identify the trends in sensory processing assessments [10] and highlighted that the most common measure to assess sensory processing is the caregiver report on Sensory Profile [11].

Although the heterogeneity of sensory dysfunctions in individuals with ASD is well known, there is still no strong consensus on both the neural mechanisms underlying the alteration of sensory processing and the ways in which these alterations affect the cognitive functioning of these individuals [12]. However, Baum and colleagues [12] highlighted as the scientific literature suggest that low-level sensory changes in ASD are likely to influence several areas of behavior such as social communication because of the important role of sensory and multisensory representations for the forming of the building blocks of higher-order cognitive representations.

In addition to sensory issues, there is increasing evidence that individuals with ASD also have motor deficits, including postural control and gait impairments [13,14,15]. Esposito et al. suggested that atypical gait patterns could be considered as early symptoms of brain abnormalities in these children since toddlers with ASD show higher asymmetry during walking when compared to children with a neurotypical development [16]. 

Among these motor signs, toe-walking is a possible clinical finding, and it is one of the earliest and most evident manifestations of a qualitative impairment in the motor domain in ASD. Toe-walking (TW) diagnosis is applied when a subject continues to walk on his toes even when a mature heel-toe gait might be achieved (i.e., 3–7 years) [17], and manifests this tendency for more than 6 months [18]. Since this behavior can be manifested also during standing or running [19,20], a more extensive term such as “tip-toe behavior” (TTB) could be used [21]. 

TTB can be observed in a spectrum of clinical manifestations, and it can present with different levels of severity both in children with typical development and in children with neurodevelopmental disorders, including ASD [22,23,24]. In some individuals with ASD, TTB can completely disappear over time without any intervention. In others it can persist with different frequencies. Some persons with ASD can exhibit TTB sometimes during the day and can perform a typical heel strike when they focus slightly on their gait; on the other hand, other persons with ASD can display TTB very often during the day and they must concentrate a lot to decrease the behavior. For some persons with ASD, TTB persistence can become a social stigma as well as a possible cause of physical problems. For example, a lot of individuals with ASD report the occurrence of calf discomfort/pain during daily life activities such as jogging. A cohort study identified the presence of three mutually exclusive classes of increasing severity in which TTB manifests itself: (1) TTB present only when running; (2) TTB present when walking and running; (3) TTB present when standing, walking, and running [21]. Moreover, a relationship between TTB severity (i.e., TTB presence during standing, walking, and running) and both Gastrocnemius and Soleus muscle shortening was found [25]. This, in turn, can facilitate a higher risk of falling [26] and influence the individual’s quality of life [27]. 

To date, the etiology of TTB is not clearly understood [18], and several hypotheses are suggested. Weber (1978) proposed that “toe walking arises from the fixation of a normal transient stage of development” [19]. Accardo suggested TTB as a “residual of a primitive reflex (i.e., positive support reflex or tonic labyrinthine reflex)” [18]. This is corroborated by Shaw and Soto-Garcia (2021) who described the decreasing of TTB after utilizing primitive reflex integration exercises (i.e., asymmetrical tonic neck reflex) [28]. Other authors suggest TTB as a result of a vestibular issue [18]. Some data from the scientific literature suggest the possibility that TTB in individuals with ASD may be caused by difficulties in sensory modulation or processing. Accardo and Barrow (2015) suggested that TTB could be a sign of a sensory modulation impairment [24]. This idea is further supported by the results of a preliminary study conducted on 14 children with ASD (7 with TTB and 7 without TTB), which showed a significant reduction of TTB during standing and walking on a soft floor surface (foam mats) compared to standing and walking on a linoleum floor [21]. Later, Wilder and colleagues found a decrease of TW when two persons with ASD walked on different surfaces [29], replicating the Fanchiang et al. study results in individuals with idiopathic toe walking (ITW) [30]. Liu (2013) explored the relationship between sensory processing (assessed using the Short Sensory Profile) and motor performance (evaluated with the Movement ABC-2), evidencing a relationship between delayed sensory processing and fine and gross motor difficulties of children with ASD [31]. 

According to these findings, assessing the sensorial patterns of ASD subjects presenting with gait abnormalities could provide useful information for programming interventions when rehabilitation for TTB in children and adolescents with ASD is necessary. The possible link between motor and sensory problems is supported in literature both in children with ASD and other developmental conditions [31,32,33], but the modalities of interconnection and the neurological substrates to these clinical features are not yet clear. Although it is widely accepted that sensory processing differences are a key point in the functioning of individuals with ASD, the study of these features is still essential to the understanding of the effectiveness and usefulness of the various therapeutic approaches. To our knowledge, no study has addressed the specific sensorial pattern of individuals with ASD and TTB and compared it with the sensorial pattern of those with ASD and NO-TTB.

In this light, the main aim of this pilot study is to explore sensory features in a sample of ASD children and adolescents with or without TTB. Our hypothesis is to find differences in sensory processing between ASD individuals with TTB and ASD individuals without TTB.

## 2. Materials and Methods

### 2.1. Sampling and Data Collection

This retrospective observational pilot study was conducted in the Autism Research Unit of “Villa S. Maria Institute”, in Tavernerio (Como, Italy). Inclusion criteria were: (i) ASD diagnosis according to DSM-5 criteria and confirmed using the Autism Diagnostic Observation Schedule; (ii) age between 7 and 18 years; (iii) individuals with ASD had to have a psychoeducational and rehabilitative management by a multidisciplinary team (child neuropsychiatrist, physiatrist, neurodevelopmental disorders therapist, psychologist, special education teacher, physical therapist, and speech therapist). For this study, specific evaluations were performed by two neurodevelopmental disorders therapists (A.N., R.P.), two physiatrists (M.M., C.P.), and one physical therapist (G.V.). Children with major sensory or neurological impairments or with genetic syndromes or with the presence of comorbid diagnosis that would have an impact on gait (i.e., individuals with cerebral palsy, upper motor neuron syndromes, neuromuscular disorders, tethered spinal cord, Down Syndrome, Rett Syndrome) were excluded. Moreover, the presence of intellectual disability was also assessed and classified as mild, moderate, severe, and profound, referring to DSM-5 criteria. Trained expert developmental psychologists performed clinical and diagnostic evaluations for ID.

An informed consent form was signed by parents or guardians of children and adolescents that participated in this study. Local IRB Insubria’s Ethics Committee gave the ethical approval (n° 250/2018). The protocol study was carried out in accordance with the Declaration of Helsinki.

Therefore, the records of 32 Italian ASD subjects (4 females; 28 males) were analyzed. Subjects were divided into two groups based on the presence or not of TTB (i.e., 16 individuals with ASD and TTB and 16 individuals with ASD without TTB). Details of the demographic and clinical characteristics of the study sample are presented in Table 1.

### 2.2. Measures 

#### 2.2.1. Short Sensory Profile

The sensory pattern was explored using the Italian cross-cultural validation of the abbreviated version of the Sensory Profile-1st version, the Short Sensory Profile 1 (SSP1) [34]. For a description of the Italian cross-cultural validation process see the Supplementary Material S1 [11,34,35,36,37,38,39,40,41], references about Supplemental Material S1. The SSP is an abbreviated form of Dunn’s Sensory Profile caregiver questionnaire (SP), created as a screening tool to identify sensory processing difficulties in children [11]. The 38-item SSP was originally derived from psychometric analyses of SP questionnaires completed by the caregivers of 117 children aged 3–17 years [42]. SSP1 is organized into seven subscales: Tactile Sensitivity (7 items), Taste/Smell Sensitivity (4 items), Movement Sensitivity (3 items), Under responsive/Seeks Sensation (7 items), Auditory Filtering (6 items), Low Energy/Weak (6 items), and Visual/Auditory Sensitivity (5 items). The SSP1 total score and the score on each subscale can be used to classify children’s level of sensory profile (Typical performance, Probable Difference, or Definite Difference) based on score percentiles from a large normative sample of children with typical development (from 3 to 10 years of age) [11]. We used the SSP1 and performed an Italian cross-cultural validation process because when we started to collect the data (in 2017) the Sensory profile—the 2second version was not yet available in the Italian language and the Sensory Profile first version was available in Italian language only for the long form. SSP1 questionnaires were filled out by the special education teachers who followed each child individually in the normal school context, according to the Italian School model; this choice also ensured greater objectivity and precision for the data collection. Despite the validation reported by the authors concerning children up to 10 years of age, we decided to administer SSP1 to school-aged subjects until 18 years of age because of their ID level, as already used by other authors [42,43,44,45,46].

#### 2.2.2. Tip-Toe Behavior Assessment

The assessment of TTB was performed through a qualitative evaluation, with the aim to establish exclusively the presence/absence of TTB during standing and/or walking and/or running. For this aim, an ecological, non-invasive, and standardized assessment previously utilized with people with ASD and ID and already described by Valagussa and collaborators [21] was utilized for making the diagnosis of TTB. The assessment was conducted with the following steps:

(1) The presence or absence of TTB during standing, walking, and running was noted by an expert physical therapist observing the spontaneous behavior of the person during daily life activities.
(2) The same therapist asked the main caregiver about the presence or absence of TTB during standing, walking, and running in daily life activities, without informing about the results of the previous observation.

If the two observations agreed, the assessment was concluded. When the observations of the therapist and the caregiver disagreed, the therapist did a standardized assessment as follows in point 3.

(3) For the final decision about the presence or not of TTB while standing, persons were invited to play while standing in front of a table once a day for 3 days. For the final decision about the presence or not of TTB during walking, persons were invited to walk three times for a distance of 10 meters on 3 separate days. For the final decision about the presence or not of TTB during running, persons were asked to run 10 meters three times on 3 separate days.

The presence of TTB was excluded when a complete absence of the behavior resulted in all three trials. No other quantitative parameters were evaluated, and the presence/absence of TTB was treated as a categorical factor (0 = absence; 1 = presence).

### 2.3. Statistical Analysis

As indicated in CORSORT guidelines for pilot and feasibility trials [47], a sample size calculation was not performed for this pilot study. The researchers aimed for 32 participants because it was felt this would be a large enough sample to inform them about the sensory features of children and adolescent with ASD and TTB and to obtain necessary preliminary information to plan further studies with a larger sample.

In this pilot study, we presented data as a percentage and mean ± standard deviation (SD) for nominal and continuous variables, respectively. Data were checked for normal distribution (i.e., Shapiro–Wilk test *p*-value > 0.05 and by visual inspection of Q-Q plot). T-test was used if assumptions for data normality and homogeneity were met. Mann–Whitney U test was used for data non-normally distributed. 

A preliminary standard statistical test was employed to check the comparability of the two groups (16 ASD TTB vs. 16 ASD NO-TTB) for age (i.e., the non-parametric Mann–Whitney U test for two independent populations). Fisher’s exact test (two-tailed) was used to determine the association between gender and TTB presence/absence and the association between ID severity and TTB presence/absence. Fisher’s exact test (two-tailed) was also used to determine the association between each of the SSP1 sections and TTB presence/absence and the association between SSP total score and TTB presence/absence.

The significance level was set at a *p*-value < 0.05. Data analysis was conducted using SPSS version 23.0 for Windows (IBM Corp., Armonk, NY, USA).

## 3. Results

### 3.1. Preliminary Statistical Analysis 

The 16 children and adolescents of ASD TTB group were matched for age and gender with 16 children and adolescents with ASD and without TTB. In addition, preliminary standard statistical tests were employed to check the comparability of the two groups (ASD TTB vs. ASD NO-TTB). The mean age value of the TTB group was 12.84 years (SD 3.04; median: 12.91 years) versus a mean age value of 13.02 years (SD 2.89; median: 13.42) in the NO-TTB group. Performing a Mann–Whitney U test, we did not find a statistically significant difference in age distribution between the two groups. 

The ID severity of the study sample was described in Table 1. Because of the results of the ID distribution, we used Fisher’s exact test to verify the association between ID severity distribution of persons with “mild + moderate” and “severe + profound” ID and TTB presence/absence. There was no statistically significant association between the two variables.

### 3.2. Differences between the Two Groups

**Figure 1:**
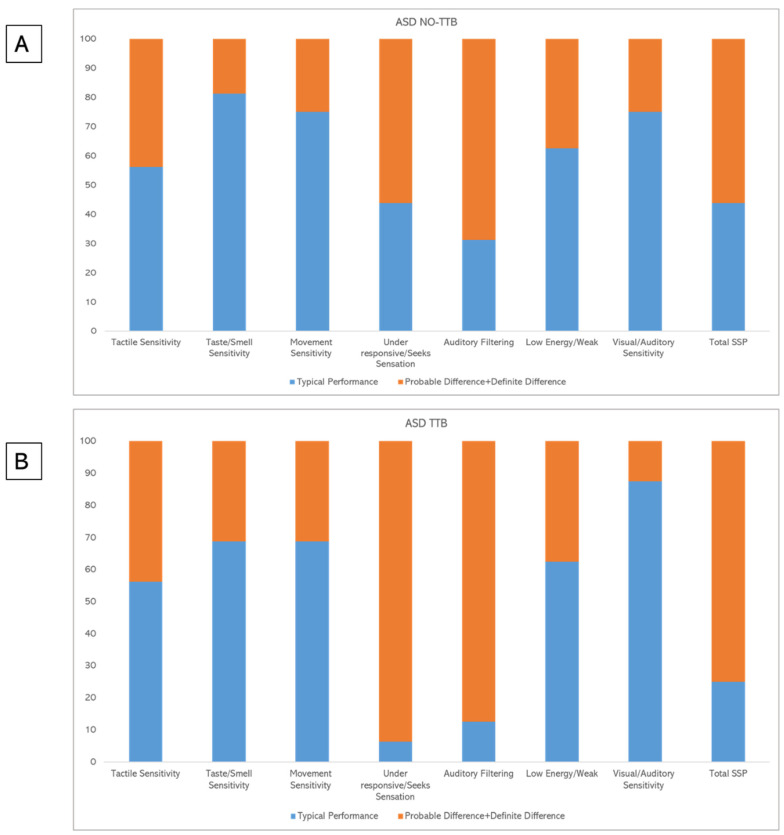
(**A**) Percentages of children that have scores in the normal or impaired ranges among the SSP1 scales in the ASD NO-TTB group. (**B**) Percentages of children that have scores in the normal or impaired ranges among the SSP1 scales in the ASD TTB group.

We found the presence of sensory impairment in 65.6% of the individuals with ASD assessed in our study, expressed as “probable difference” or “definite difference” at SSP total score. Performances at the SSP sections were presented in Table 2 for NO-TTB and TTB groups. We found that 25% (*n* = 4) of the NO-TTB participants obtained a “definite difference” in the SSP total score, while the same score as “definite difference” was obtained by the 50% (*n* = 8) of the TTB subjects (see Table 2 and Figure 1). 

The data of several SSP1 sections showed that the highest number of subjects with a “definite difference” in the ASD TTB group was in the “under-responsive/seeks sensation” (81.3%; *n* = 13) and the “auditory filtering” (56.3%; *n* = 9) sections. As reported in Figure 1, these two sections of the SSP1 are the most compromised in the TTB group and the distribution of these sensory dysfunctions between the two groups appear very different.

We used Fisher’s exact test to determine the association between the distribution of subjects with “typical performance” versus “probable difference + definite difference” group in both SSP1 total score and in each of the SSP1 sections and TTB presence/absence. We found a statistically significant association between the two variables in the “under-responsive/seek sensation” section (two-tailed *p* = 0.037). For details of each test result, see Table 3.

## 4. Discussion

To our knowledge, this is the first study comparing sensory features of ASD children and adolescents with or without TTB, utilizing SSP1, a commonly used parent-report questionnaire for the identification of sensory processing differences in children. The main result is summarized as follows: both ASD groups tend to present significant sensory-related behavioral symptoms, but the ASD TTB group showed more frequently the specific pattern of “under-responsive/seeks sensation” than the ASD NO-TTB group.

The sensory-related behavioral symptoms of our ASD sample are similar to the results of previous studies [31,48,49]. Furthermore, Purpura and collaborators [33] suggested the presence of early sensory-motor abnormalities in children with ASD and the link between sensory dysfunctions and motor abnormal behaviors in this population already during the preschool age. This finding is line also with data of Uljarevic’ and colleagues that highlighted the predictive role of early motor development and of motor atypicalities, for example toe walking, on social responsiveness abilities and on the severity of ASD symptoms [50]. 

Interestingly, Williams et al. (2014) investigated the differences between sensory processing capacity in 60 typical children (age range: 4–8 years) who manifested and non-manifested an ITW using the parent-report Sensory Profile [51]. They found a statistically significant difference in the sensory profile of ITW when compared to children with a normal gait and, similarly to our findings, “Sensory Seeking” mean scores were noted as being ranked as a ‘‘more probable difference’’ when compared to “normative population” [51]. Recently, Chu, Girolami & Grant-Beuttler assessed sensory differences in 10 individuals with ITW versus an age-matched control group [52] using a battery of measurements: motor assessment (i.e., Test of Gross Motor Development 2nd edition), sensory modulation assessment (i.e., Sensory Processing Measure, electrodermal activity response to sensory stimuli, response to vestibular stimulus), tactile processing assessment (i.e., light touch perception threshold, vibration perception, gait on different surfaces), balance assessment (i.e., NeuroCom^®^ SMART Balance Master^®^ Sensory Organization Test and Adaptation Test), and an ankle proprioception test [52]. They found a statistically significant sensory modulation deficit in the ITW group. Seven out of 10 individuals had statistically significantly higher than normal resting electrodermal activity, 5 out of 10 showed a hyposensitivity to the vibration perception threshold test, and 3 out of 10 showed a hyposensitivity to the monofilaments test. All these results support an impairment of sensory processing that can be expressed in different ways. 

Thus, the sensory seeking profile is present in individuals with ASD who exhibit TTB as well as in individuals with ITW, but data about the etiology of this motor symptom in individuals with ASD are still scarce and conflicting. According to the literature, the sensory seeking’s behavior seems not to be specific to individuals with ASD and this would suggest that the ASD individuals and no-ASD individuals shared some processes but also differed in other predictors of sensory-motor development. Indeed, the finding of an “under-responsive/seek sensation” pattern in ASD TTB group seems to be in contrast with the common belief that TTB is a behavior manifested in response to an oversensitivity of the sole of the foot. A lower tactile threshold was found in children with ASD when compared with age-matched controls [53]. Also, a lower vibrotactile detection threshold was found in ASD children at the hand [54]. The same results were found by Williams et al. [55] when they assessed vibrotactile detection thresholds of the hallux in 30 typical children with ITW compared to 30 age-matched controls (4–8 years). Conversely, Ganley and Behnke [56] assessed vibration perception threshold at the metacarpal and metatarsal phalangeal joints in 11 toe walkers and 15 age-matched controls (age range 4–12) and found that mean vibratory threshold was higher in toe walkers compared with controls at both sites. The differences could be explained by differences in the populations studied. Another possible reason could be the presence of two distinct and opposed mechanisms that produce TTB: some subjects may express TTB to increase sensory input (e.g., TTB could be a mechanism to increase the muscle tone of the lower limb, and thus could be used as a strategy to improve the perception of the limb), while others to decrease undesired stimulation to all the foot area (e.g., tactile inputs), as also previously suggested by Ganley and Behnke [56] and later by Chu et al. [52]. This can fit with the complexity that ASD represents [57], as also with the complexity of the sensorimotor system [58]. From this perspective, as suggested from different authors [59,60], the ASD could be considered as a form of human neurodiversity that manifests in a set of strengths and difficulties in sensory-motor processing that may differ to the typical population.

With regard to therapeutic possibilities and indications, a recent systematic review summarized the evidence on conservative, pharmacological, and surgical interventions for TW in individuals with ASD [61]. The authors included nine papers, all with a case study—case report study design. They noted that all included studies were addressed to evaluate conservative intervention effectiveness. Serial casting +/− orthoses and behavioral interventions (i.e., using acoustic feedback associated with positive reinforcement, or a wristband as a discriminative stimulus) were the most studied interventions. Moreover, despite pharmacological and surgical interventions being used to treat TTB in persons with autism in clinical practice [62], no studies that assess effectiveness of pharmacological and surgical interventions were found [61]. 

The systematic review results also highlighted that, at this moment, the proposed treatments for TTB tend to be symptom-based rather than etiology-based, as also previously recognized in a commentary by Bhat and Kaznica [63]. This confirms the necessity to conduct studies aiming to understand the etiology of TTB. From this perspective, further studies going deeper into sensorimotor processes at the basis of TTB would be relevant. If the interlink between sensory issues and TTB should be verified in ASD individuals, this could guide clinicians on the use of ecological and non-invasive therapies, based on the sensory integration approach. 

Moreover, although TTB could be the expression of individual differences in sensory activation thresholds, because TTB can express itself with different severity trajectories, the prolonged use of TTB in childhood might have consequences with a negative impact on the quality of life of these patients [26,27]. Thus, a standardized tool to quantitatively assess TTB in subjects with ASD would be useful to monitor TTB trend [64]; this would permit planning a timely treatment before muscle length shortening develops. 

Based on these findings, it is necessary to reflect on the importance of the early sensory-motor assessment and intervention for preventing toe-walking, not only for the key-role of sensory processing in motor learning and adaptation, but also to avoid the negative impact of motor dysfunctions on the quality of life of these children.

Finally, our study design does not allow us to state if sensory issues contribute to TTB or if sensory impairment is the result of persistent TTB. Therefore, further studies might hopefully address this issue.

## 5. Limitation

The authors recognize two main limitations of this study. The first refers to the study design based on the retrospective analysis of data. The second is the limited sample size, making our results preliminary. If on the one hand, we are inclined to consider very carefully the sensory differences between TTB and NO-TTB ASD subjects, on the other hand, we feel that the study results are very promising and might be confirmed using prospective study designs. We believe that the limitations presented by its small sample size and retrospective designs are outweighed by its originality. 

Thus, consequently to these reflections, it could be interesting to conduct a larger study on subjects with ASD with and without TTB, comparing results of both the caregiver questionnaire (specifically with the new validated version of SP2 or others new tools as Sensory Processing Measure—SPM) and more specific objective measures (i.e., proprioception and/or two-point discrimination and/or touch perception threshold and/or vibration threshold). Moreover, new insights could be gained by the conduction of studies to compare subjects with ASD and TTB versus ITW groups to assess the presence of a common underlying mechanism or of mechanisms specific to a single subgroup.

## 6. Conclusions

The study findings showed statistically significant differences in the sensory profile between children and adolescents with ASD and TTB and children and adolescents with ASD but without TTB. In particular, ASD subjects with TTB showed more frequently the specific pattern of “under-responsive/seeks sensation” than ASD NO-TTB individuals. These preliminary findings, in accordance with the literature, support the importance of considering the sensory aspects when a rehabilitative approach is planned to treat persons with ASD and TTB, and also the clinical monitoring of people who manifest a severe TTB because of the potential negative effect on the quality of life of this population. Finally, an early and ecological approach based on sensory integration could be an important instrument to prevent toe-walking and other motor dysfunction in children with ASD.

## Figures and Tables

**Table 1:** Main demographic and clinical characteristics of the study sample.

Demographic and Clinical Findings	Total ASD Sample (N. 32)	ASD NO-TTB Group (n. 16)	ASD TTB Group (n. 16)
Mean Age (SD) (yrs); Age median (yrs)	12.93 (2.92); 13.16	13.02 (2.89); 13.42	12.84 (3.04) 12.91
Age range (yrs)	7.31–17.88	7.44–17.51	7.31–17.88
Gender (M/F)	28/4	14/2	14/2
Mean ADOS-2 CSS (SD); median ADOS-2 CSS range	7.56 (1.63); 7 4–10	6.75 (1); 7 5–9	8.38 (1.75); 8.5 4–10
ID Mild n. (%)	1 (3.125%)	0	1 (6.25%)
ID Moderate n. (%)	1 (3.125%)	0	1 (6.25%)
ID Severe n. (%)	12 (37.50%)	11 (48.49%)	1 (6.25%)
ID Profound n. (%)	18 (56.25%)	5 (45.46%)	13 (81.25%)

Legend. ASD = autism spectrum disorder; NO-TTB = no tip-toe behavior; TTB = tip-toe behavior; CSS = calibrated severity score; ID = intellectual disability.

**Table 2:** Performance on the SSP for NO-TTB and TTB groups.

Section	Class	NO-TTB n° 16	TTB n° 16
Tactile Sensitivity—n° (%)	Typical performance	9 (56.2%)	9 (56.2%)
	Probable difference	1 (6.3%)	2 (12.5%)
	Definite difference	6 (37.5%)	5 (31.3%)
Taste/Smell Sensitivity—n° (%)	Typical performance	13 (81.3%)	11 (68.8%)
	Probable difference	1 (6.3%)	2 (12.5%)
	Definite difference	2 (12.5%)	3 (18.8%)
Movement Sensitivity—n° (%)	Typical performance	12 (75%)	11 (68.8%)
	Probable difference	2 (12.5%)	1 (6.3%)
	Definite difference	2 (12.5%)	4 (25%)
Under responsive/Seeks Sensation—n° (%)	Typical performance	7 (43.8%)	1 (6.3%)
	Probable difference	2 (12.5%)	2 (12.5%)
	Definite difference	7 (43.8%)	13 (81.3%)
Auditory Filtering—n° (%)	Typical performance	5 (31.3%)	2 (12.5%)
	Probable difference	5 (31.3%)	5 (31.3%)
	Definite difference	6 (37.5%)	9 (56.3%)
Low Energy/Weak—n° (%)	Typical performance	10 (62.5%)	10 (62.5%)
	Probable difference	0	0
	Definite difference	6 (37.5%)	6 (37.5%)
Visual/Auditory Sensitivity—n° (%)	Typical performance	12 (75%)	14 (87.5%)
	Probable difference	4 (25%)	2 (12.5%)
	Definite difference	0	0
Total SSP—n° (%)	Typical performance	7 (43.8%)	4 (25%)
	Probable difference	5 (31.3%)	4 (25%)
	Definite difference	4 (25%)	8 (50%)

Legend. SSP = short sensory profile; TTB = tip-toe behavior; NO-TTB = no tip-toe behavior.

**Table 3:** Fisher’s exact test results for each section and SSP total score.

	SSP Total Score		Tactile Sensitivity		Taste/Smell Sensitivity		Movement Sensitivity		Under-Responsive/Seek Sensation		Auditory Filtering		Low Energy/Week		Visual/Auditory Sensitivity	
	TTB	NO TTB	TTB	NO TTB	TTB	NO TTB	TTB	NO TTB	TTB	NO TTB	TTB	NO TTB	TTB	NO TTB	TTB	NO TTB
**TP n° subjects**	4	7	9	9	11	13	11	12	1	7	2	5	10	10	14	12
**PD + DD n° subjects**	12	9	7	7	5	3	5	4	15	9	14	11	6	6	2	4
**Fisher’s exact test (two-tailed)**	*p* = 0.458		*p* = 1		*p* = 0.685		*p* = 1		*p* = 0.037 *		*p* = 0.394		*p* = 1		*p* = 0.654	

Legend. SSP = short sensory profile; TP = typical performance; PD + DD = probable difference + definite difference. * = statistically significant.

## Data Availability

The data that support the findings of this study are available from the corresponding author [CP] upon reasonable request.

## Supplementary Materials

The following supporting information can be downloaded at: https://www.mdpi.com/article/10.3390/children9091336/s1, Supplementary Material S1.
