# Supplementary material for: Sensory Profile of Children and Adolescents with Autism Spectrum Disorder and Tip-Toe Behavior: Results of an Observational Pilot Study
Source: Children (Basel). 2022 Sep 1;9(9):1336. doi: 10.3390/children9091336 (PMC9497722; doi:10.3390/children9091336)
Supplement: Supplementary file 1 [file children-09-01336-s001.zip › children-1883947-supplementary.pdf]

Supplementary material 1

Sensory profile of children and adolescents with autism spectrum disorder and tip-toe behavior: results of an observational pilot study

Cross-cultural adaptation of the Italian version of the Short Sensory Profile

METHODS

Short Sensory Profile – First Edition

The Short Sensory Profile – First Edition (SSP1) is a questionnaire used to screen children aged 3–10 based on caregiver reports. It is divided into 7 sections: Tactile Sensitivity (7 items), Taste/Smell Sensitivity (4 items), Movement Sensitivity (3 items), Under-responsive/Seeks Sensation (7 items), Auditory Filtering (6 items), Low Energy/Weak (6 items), and Visual/Auditory Sensitivity (5 items). The frequencies of engagement in the described behaviors are indicated on a 5-point Likert scale (1 = always, 2 = frequently, 3 = occasionally, 4 = seldom, and 5 = never) [34]. The total score varies from 190 to 38. Scores from 190 to 155 are interpreted as "typical performance". A score ranging from 141 to 38 is categorized as "definite difference" indicating the likelihood that the child is having significant problems with processing sensory stimuli and consequent difficulties in performing daily life activities. An intermediate score ranging from 154 to 142 is classified as "probable difference" [11]. Table S1 describes in detail the score range of each of the three categories for the 7 sections. According to the developers of the questionnaire, the internal consistency and internal validity of the total score and the individual section scores range from 0.70 to 0.90 and 0.25 to 0.76 respectively. The SSP can be used to screen for a variety of sensory processing difficulties due to the conceptual relationship between sections of SSP and the long Sensory Profile [34].

Table S1: Short Sensory Profile scores

SSP sections	Typical performance score	Probable difference score	Definite difference score
Tactile Sensitivity	35 - 30	29 - 27	26 - 7
Taste/Smell Sensitivity	20 - 15	14 - 12	11 - 4
Movement Sensitivity	15 - 13	12 - 11	10 - 3
Under-responsive/Seeks Sensation	35 - 27	26 - 24	23 - 7
Auditory Filtering	30 - 23	22 - 20	19 - 6
Low Energy/Weak	30 - 26	25 - 24	23 - 6
Visual/Auditory Sensitivity	25 - 19	18 - 16	15 - 5
Total SSP score	190 - 155	154 - 142	141 - 38

Translation and Cross-Cultural Adaptation

After obtaining permission from Pearson [35] we proceeded to translate the instructions, items, and summary sections contained in SSP, that at the beginning of our study were not available yet in the Italian language.

Step 1: The first step was a forward translation from English to Italian. Two versions (named T-1 and T-2) were independently produced by two bilingual native Italian translators. Both the two translators (N.A. and P.R.) were neurodevelopmental disorder therapists with 10-years of clinical experience with patients and with a linguistic high school degree. All original items, instructions and summary sections were translated.

Step 2: The two versions were then synthesized into one single translation by consensus (T-12). Discrepancies in the wording choices were identified and resolved by discussion between the two translators.

Step 3: Two bilingual, native English translators (fluent in Italian) independently backward translated the T-12 version into the original language (BT-1 and BT-2) to check gross inconsistencies or conceptual errors in the translations. One translator was a physiotherapist with 40-year clinical experience but without clinical experience with pediatric patients, the other had a bachelor's in social cultural anthropology and a Master in Education. They had no previous knowledge of SSP.

Step 4: Expert committee. The expert committee included 8 people: one expert methodologist, two health professionals, a language professional, and the four translators (forward and back translators) involved in the process. The expert methodologist was a doctor with a specialization in gastroenterology and with 40 years of research experience, and the language professional was a professional translator with 15 years of experience. The two health professionals were a physical therapist with 24-year clinical experience with pediatric patients and a Clinical Psychologist with 10 years of clinical experience with pediatric conditions. The committee reviewed all the translations and reached a consensus on any discrepancies. The material at the disposal of the committee included the original questionnaire, and each translation (T-1, T-2, T-12, BT-1, BT-2) and long form of SP1. The committee examined the source and back-translated questionnaires for all semantic, idiomatic, experiential, and conceptual equivalences and in case of doubts or disagreement for some items, it was taken into consideration the same items of the long version of the Italian SP1. As requested in the research translation license agreement, a clean copy of the Translated Test was provided to Pearson before use in the Research Project, together with a back translation into English and an explanation of any departures from a literal translation.

Step 5: Testing of the SSP-I version. Pilot testing of the SSP-I was conducted on 11 Italian caregivers of ASD subjects.

Content Validation by Neurodevelopmental Disorders Therapist

This process was carried out by 9 Neurodevelopmental Disorders Therapists proficient in Italian with 6–13 years of working experience in the field of pediatrics to further support the conceptual equivalence (clarity) of the SSP-I. They were asked to rate the instructions, items, and the response format of the SSP-I as "clear" or "unclear" and to provide suggestions to improve the clarity of statements that were rated as "unclear."

The same nine therapists were also asked to examine the content validity of the SSP-I by using a 4-point rating scale (1 = "not relevant" to 4 = "very relevant") [56] and evaluate the relevance of individual items of the SSP-I. An expert panel of 3–10 members is suggested by Lynn (1986) [37].

Data Analysis

The minimum inter-rater agreement among the experts for clarity assessment was set at 80% [38]. Content validity examines the degree to which samples of items, taken together, constitute an adequate operational definition of a construct being measured [39]. The assessment of content validity by the panel of experts was analyzed by calculation of the content validity index (CVI). The CVI is easy to understand and can be used as a guide for the modification or deletion of instrument items [40]. The CVI was calculated at both item level (I-CVI) and scale level (S-CVI). The I-CVI is computed as the number of experts giving a rating of 3 or 4 to the relevance of each item, divided by the total number of experts. The I-CVI which expresses the proportion of agreement on the relevance of each item should be at least 0.78 [39,40]. The S-CVI was defined as "the proportion of items on an instrument that achieved a rating of 3 or 4 by the content experts" [37,39,40]. The S-CVI which was calculated using the average calculation method, S-CVI/Ave should be ≥ 0.90 [41].

RESULTS

Translation and Cross-Cultural Adaptation

The forward translation process was carried out without much difficulty. Making a comparison between T1 and T2 versions, there were no significant differences between them in both the instruction and item sections. In five items the two translators reached an agreement about the translation of single words.

Also, the back-translation process was carried out without much difficulty. Only in item 21, the back translation evidenced the difficulty to translate the word "twisted" using a single word. For this reason, after a discussion, the expert committee decided to translate this word using the Italian expression "lascia in disordine". The expert committee examined the source and back-translated questionnaires for all semantic, idiomatic, experiential, and conceptual equivalences. Based on recommendations by this expert panel, minor modifications were made to the draft Italian version to produce an amended version, the SSP-I.

Content Validity

Inter-rater agreement among the 9 experts for all the item's clarity ranged from 89% to 100%. The CVI for 38 items ranged from 0.78 to 1.00 and S-CVI/Ave was 0.95. In the content-related validation by 11 caregivers for clarity of the SSP-I, no major changes were proposed by the participants regarding clarity.
